# Generic group metacognitive therapy for depression in specialized mental health care: an open trial with 6- and 12-month follow-ups

**DOI:** 10.3389/fpsyt.2025.1704076

**Published:** 2026-01-08

**Authors:** Henrik Nordahl, Erlend Pukstad, Odin Hjemdal, Lise T. Veium, Eivind R. Strand

**Affiliations:** 1 St. Olavs Hospital, Trondheim University Hospital, Nidaros DPS Section 1, Trondheim, Norway; 2 Department of Psychology, Norwegian University of Science and Technology, Trondheim, Norway

**Keywords:** CAS, comorbidity, cost-effectiveness, group, health economics, major depressive disorder, metacognitive therapy, transdiagnostic

## Abstract

**Introduction:**

Depression is the most prevalent mental disorder and a common cause for seeking treatment in specialist mental health care settings. Along with having a broad negative impact on general functioning, depression is frequently comorbid with other mental disorders and can, in many presentations, be complex to treat, with substantial relapse rates following treatment. Metacognitive therapy (MCT) aims to modify common determinants of distress to improve mental regulation, making it a transdiagnostic treatment well suited for delivery in a group format.

**Methods:**

In the present study, we evaluated the treatment effects associated with the generic group MCT in a Norwegian Specialist Mental Health Service for patients in need of treatment for depression. Diagnostic evaluation was conducted by independent assessors pre- and post-treatment, and patients self-reported on a range of measures pre-, post-treatment, and at six- and 12-month follow-ups.

**Results:**

Twenty patients divided into four groups received group MCT delivered by two therapists over 10 sessions. The treatment was associated with an 80% recovery rate from depression and large effects across primary and secondary outcomes, including return to work. From post-treatment through the six- and twelve-month follow-ups, the improvements were largely maintained, but a few patients reported a clinically significant increase in depression symptoms. A health economic evaluation indicated that the treatment was cost effective.

**Discussion:**

These findings suggest that generic group MCT is well-suited for specialist mental health care services aiming to treat depression cost-effectively.

## 1 Introduction

Depression is the leading reason for seeking treatment in Norwegian specialized mental health care services (1). Those offered treatment typically have severe presentations marked by repeated depressive episodes, comorbidity, non-response to previous treatment or relapses, and sometimes, ambivalence to change (e.g., 2). Heterogeneous and complex presentations in patients with depression must be expected given the high comorbidity with anxiety disorders (3), personality disorders (4), and association with short- and long-term sick leave (5). Unfortunately, limited recovery and high relapse rates are the norm rather than the exception for patients receiving usual care (6). Therefore, specialist mental health care services require innovative treatment interventions that effectively reduce the totality of patients’ symptoms and functional impairments in the long term. In addition, to be viable, interventions must be cost-effective, considering limited healthcare resources.

The metacognitive model (7, 8) and therapy (9) are interesting in this regard for several reasons. It is based on the principle that psychological disorders are caused by a *Cognitive Attentional Syndrome* (CAS) consisting of a perseverative and negative thinking style, strategic and rigid attentional focus on threatening information, and maladaptive behavioral strategies meant to compensate for poor mental regulation. The CAS is a transdiagnostic feature directed by underlying biases in metacognition (i.e., knowledge and plans applied to cognition, e.g., “rumination is uncontrollable”). These mechanisms may explain both comorbidity and symptom overlap between various emotional and psychological problems, in addition to the recurrent and persistent nature of depression (10). For example, metacognitive beliefs about the uncontrollability of negative thinking (i.e., the CAS) can intensify and maintain anxiety and depression, self-esteem issues, interpersonal problems, and poor work ability (11–16). Metacognitive therapy (MCT) was developed and tailored to effectively reduce CAS and modify biases in metacognition (9). Based on the understanding that disorders are characterized by common underlying mechanisms (i.e., CAS and biases in metacognition), a typical dosage of eight to 12 sessions is used to achieve adequate metacognitive change in emotional disorders. Furthermore, as the therapeutic focus and interventions are concerned with similarities (e.g., repetitive negative thinking and metacognitive beliefs about these thinking styles) rather than the differences between disorders and presentations (e.g., self-beliefs, background), MCT is well-suited to be delivered in a group format, which is also considered a cost-effective option due to the therapist-per-patient ratio.

Meta-analyses indicate that MCT is effective and potentially more effective than other psychological treatments commonly used to treat anxiety and depression (17, 18). For patients with depression, individual MCT is superior to wait-list (19) and cognitive behavior therapy (20). It is further associated with positive effects in comorbid cases (21), persistent/chronic presentations (22), and improved neuropsychological function (23). Two studies have evaluated disorder-specific MCT for depression delivered in a group format and reported encouraging results (24, 25).

The aforementioned studies evaluating MCT for depression have used a disorder-specific approach that captures unique processes and specific metacognitive content in their maintenance, which enables high specificity. In a specialist mental healthcare setting, a generic/transdiagnostic (i.e., not disorder-specific) group MCT may be a good treatment approach compared to diagnosis-specific treatment. At the cost of lower specificity, it enables more room to include relevant metacognitive processes and content, regardless of diagnosis and comorbidities. Hence, it provides a balance between sufficient specificity in addressing universal metacognitive mechanisms of change (i.e., uncontrollability beliefs relevant to psychological disorders in general according to the metacognitive model) and individual differences in concurrent comorbidities, such as anxiety, low self-esteem, and personality difficulties, which may be linked to different elements of the CAS. Patients seeking treatment for depression in these settings often present with a history of repeated depressive episodes, ambivalence to change, non-response to previous treatments, and a high level of comorbidity (e.g., 26). In addition to uncontrollability beliefs and other typical metacognitive mechanisms involved in depression (9), usual biases in metacognitive beliefs among more complex patients include believing that the mind/brain is damaged or defective, that holding on to negative cognitions protects them from prolonged negative affect or interpersonal loss, and dysfunctional metacognitive goals that interfere with disengagement from the CAS (8, 10). These somewhat more complex dynamics of dysfunctional metacognition can account for observed features such as resistance to change, ambivalence, self-sabotage, and low motivation to engage in the therapeutic process (8). Thus, taking a generic approach can accommodate heterogeneity in the individual dynamics of dysfunctional metacognitive beliefs and resulting CAS strategies and their short- and long-term effects on distress/dysfunction and psychological vulnerability (9).

Previous studies have evaluated transdiagnostic group MCT in different settings (e.g., private practice) and samples selected based on self-reports of anxiety and depression. They reported positive effects across symptom domains (27, 28). In a previous study, our research group (29) conducted a pilot study on generic group MCT with a pre-post design in a sample of heterogeneous patients with primary depression in a specialist mental health setting, where the treatment was feasible and associated with positive outcomes. However, while the setting and patient group were similar to the current study, the study was a pilot and left several questions unanswered, including independent clinical assessment pre- and post-treatment and follow-up assessment points. Therefore, the aim of the current study was to further evaluate generic group MCT in a sample of heterogeneous patients with primary depression. In addition to previous studies on group MCT and with the aim of assessing potential transdiagnostic effects at the disorder level, independent assessments of diagnoses were included in the pre- and post-treatment phases. Furthermore, a range of self-report measures, including emotional disorder symptoms, self- and interpersonal problems, metacognitive beliefs, and work-related variables, were used. Follow-ups at six- and 12-month post-treatment were included to evaluate the stability of improvements for one year after treatment, as patients with depression in this type of service tend to relapse (6). We hypothesized that generic group MCT would lead to significant reductions in depressive and comorbid disorders and symptoms, improved functioning, and sustained effects at 6 and 12 month follow-ups. In addition, a preliminary health economic evaluation is also reported to address the question of the cost-effectiveness of generic group MCT for depression in specialist mental health care beyond the average number of therapist sessions to treat each patient.

## 2 Methods

### 2.1 Design and procedure

The study was conducted in a naturalistic treatment setting at a specialized mental health outpatient clinic in Trondheim, Norway. A total of 21 patients were included and allocated to four treatment groups comprising, six, five, six, and four patients. All patients had a primary diagnosis of major depression warranting treatment priority and provided informed consent to participate in an open trial assessing the effects associated with generic group MCT in a “real-world clinical setting,” with six- and 12-month follow ups. This study was approved by the Regional Committees for Medical and Health Research Ethics (reference number: 494879).

Patients were referred to the outpatient clinic by their general practitioner with major depression described as their primary problem before being evaluated by the hospital intake team and assigned to a therapist for the first assessment according to standard procedure. An independent assessor (clinical psychologist) then conducted a thorough diagnostic assessment, including the SCID-5-CV (30), SCID-5-PD (31), and self-report measures. All assessments and diagnoses were quality assured by a team of clinicians, with at least one specialist in clinical psychology/psychiatry present. Post-treatment, the same independent assessor who evaluated the patient at pre-treatment re-evaluated the diagnoses.

The following inclusion criteria were set to be eligible for participation: 1) signed written informed consent obtained prior to participation, 2) diagnosed with major depressive disorder (DSM-5, 32) as their primary diagnosis, 3) a minimum score of 10 on the Patient Health Questionnaire (PHQ; 33) and further a minimum score of 2 on either item one or two indicating a depressive episode, and 4) 18 years or older. The exclusion criteria were: 1) the patient cannot participate at the scheduled times for the treatment, 2) known somatic diseases in need of medical treatment or that could interfere with treatment response, 3) psychosis, 4) current acute suicide intent or in need of special treatment/assessment, 5) cluster A or B personality disorders, 6) developmental disorders, 7) substance dependence, 8) patients not stable (just started or quit) or willing to remain stable on psychotropic medication during the trial, and 9) patients with cognitive disabilities/disorders (such as dementia).

### 2.2 Participants

One patient dropped out after session two due to acute somatic disease which required immediate treatment hindering further attendance to mental health services. This patient was excluded from the data set as the drop-out was in no way related to the current treatment or research.

All the remaining participants completed the treatment. Of the 20 completers, nine (45.0%) were female, and their ages ranged from 18 to 62 (*M* = 30.1, *SD* = 11.7). The average number of diagnoses per patient was 2.25 (*SD* = 0.75), where three presented with symptoms that met the criteria for a depressive disorder as their sole diagnosis, 12 criteria for two diagnoses, four criteria for three diagnoses, and one the criteria for four diagnoses. Fifteen patients (75.0%) had recurrent depressive disorder (current episode moderate), and five (25.0%) had a moderate depressive episode. Regarding comorbidity, the most common Axis I disorder was generalized anxiety disorder (*n* = 5), followed by social anxiety disorder (*n* = 4). One patient was diagnosed with an unspecified eating disorder, one with obsessive-compulsive disorder, and one with attention deficit disorder that had been stabilized with medication over several years. On Axis II, the most common comorbid disorder was obsessive-compulsive personality disorder (*n* = 7), followed by avoidant personality disorder (*n* = 5) and mixed personality disorder (avoidant and obsessive-compulsive personality disorder) (*n* = 1). Only two patients had not received any previous psychological treatment, while all others had between one and three previous treatment attempts during adolescence and adulthood. Seven patients were on stable doses of antidepressant medications, and one patient was on a stable dose of methylphenidate for attention-deficit disorder. Regarding civil status, 11 were single, five were cohabitants, two were married, and two were in a relationship. Regarding work status before treatment, 13 were either partially (*n* = 6) or completely (*n* = 7) on sick leave due to mental health problems, and one patient (*n* = 1) received a work assessment allowance. Six were either students (*n* = 4) or working (*n* = 2) all of whom reported significantly impaired capacity and functioning in work and school activities.

### 2.3 Self-report measures

Several self-report measures were administered at pre-treatment, post-treatment, and at six- and 12-month follow-ups. The primary outcomes were independent clinical reassessment (including the SCID-5CV and SCID-5-PD) and PHQ-9. Secondary outcomes consisted of several symptoms and functional measures, as well as metacognitive beliefs.

The Patient Health Questionnaire (PHQ-9; 33) consists of nine items and measures the presence and severity of depressive symptoms over the past two weeks. Items are rated on a four-point scale from 0 (“Not at all”) to 3 (“Nearly every day”), with the total score ranging from 0 to 27, with higher scores reflecting greater depression severity.

The Generalized Anxiety Disorder 7 (GAD-7; 34) consists of seven items that assess the presence and severity of generalized anxiety symptoms over the past two weeks. Items are rated on a four-point scale from 0 (“Not at all”) to 3 (“Nearly every day”). The total score ranges from 0 to 21, with higher scores reflecting greater severity of generalized anxiety symptoms.

The Beck Anxiety Inventory (BAI; 35) consists of 21-items measuring the severity of physical and cognitive anxiety symptoms experienced in the past week. Items are rated on a four-point scale from 0 (“Not at all”) to 3 (“Severely”), with the total score ranging from 0 to 63, and higher scores reflecting higher anxiety symptoms.

The Social Interaction Anxiety Scale (SIAS; 36) is a 20-item measure that evaluates fear and responses to different social interactions. Each item is rated on a five-point scale ranging from 0 (“Not at all characteristic or true of me”) to 4 (“Extremely characteristic or true of me”). The total score ranges from 0 to 80, with higher scores indicating greater anxiety related to social interactions.

The Inventory of Interpersonal Problems (IIP-32; 37) is a 32-item measure of interpersonal problems and functioning (i.e., dysfunctional interpersonal behavior that people think they do too much, or interpersonal behavior that they find hard to do). Each item is rated on a five-point scale from 0 (“Not at all”) to 4 (“Very”). In the current study, we used the mean score across all items to capture the general interpersonal problem and dysfunction/distress severity factor, with a higher score indicating greater interpersonal distress.

The Work and Social Adjustment Scale (WSAS; 38) consists of five items that measure levels of impairment related to work, home management, social leisure, private leisure, and close relationships. All items are rated on a nine-point scale ranging from 0 (“Not at all”) to 8 (“Very Severely”). The scale ranges from 0 to 40, with higher scores indicating greater functional impairment.

The Rosenberg Self-Esteem Scale (RSES; 39) assesses global self-esteem with 10 items rated on a four-point scale from 0 (“Strongly disagree”) to 3 (“Strongly agree”). The RSES total score ranges from 0 to 30, with higher scores reflecting higher self-esteem.

The Metacognition Questionnaire-30 (MCQ-30; 40) consists of 30 items that measure dysfunctional metacognitive beliefs. The questionnaire has five subscales: positive beliefs about worry (POS), negative beliefs about the uncontrollability and danger of worry (NEG), cognitive confidence (CC), beliefs about the need to control thoughts (NC), and cognitive self-consciousness (CSC). Each item is rated on a four-point scale ranging from 1 (“Do not agree”) to 4 (“Agree very much”), with higher scores indicating stronger endorsement of dysfunctional metacognitive beliefs. The subscale scores range from 6 to 24, while the combined total score ranges from 30 to 120. Higher scores indicate greater levels of dysfunctional metacognition.

The EuroQol five dimensions with five levels (EQ-5D-5L; 41) is a five-item scale that assesses health-related quality of life. The five dimensions are mobility, self-care, usual activities, pain/discomfort, and anxiety/depression. Across the five dimensions, there are five levels of responses (1–5), where a higher score indicates more severe problems. The measure also includes a visual analog scale that assesses the patients’ overall experience of health on a scale ranging from 0 (worst health) to 100 (best health). Systematic reviews have indicated good psychometric properties (42), and Norwegian norm data have been published (43).

The Work Ability Scale (WAS) is a single item from the Work Ability Index (WAI; 44, 45) that evaluates participants current perceived work ability relative to their lifetime best. The WAS is rated on an 11-point scale ranging from 0 (“cannot currently work at all”) to 10 (“work ability at its best”); thus, a higher score reflects a better perceived work ability.

The Client Satisfaction Questionnaire (CSQ-8; 46) assesses patient satisfaction with the healthcare services received. The questionnaire consists of eight items rated on a four-point scale (e.g., “Have the services you received helped you deal more effectively with your problems?”). The total score ranges from 8 to 32, with higher scores indicating greater satisfaction. The CSQ-8 was administered only post-treatment.

### 2.4 Group metacognitive therapy

Metacognitive therapy (MCT) manuals have been developed for various disorders, including MDD, along with a generic treatment plan (9, pp.250–255). The latter approach can be used to address comorbid or heterogeneous symptom presentations and was therefore selected for the current study. The generic MCT approach is taught as part of the MCT Masterclass Level II at the MCT Institute^®^. Therapists must adeptly identify and modify the most relevant mechanistic metacognitive factors for the individual, which we aim to achieve in a group setting. More details about the specific approach used in the current trial are provided by Strand et al. (29). The treatment comprised 10 weekly sessions with a 90-minute duration (2 min × 45 min and a 15-minute break). Three clinical psychologists (the first, second, and last authors) provided the treatment. HN and ERS are accredited as MCT-I^®^ registered level II therapists, while EP is under training to become a level-I MCT-I^®^ registered therapist. HN and ERS conducted the first three groups, and HN and EP conducted the last group.

The number of therapist hours required to treat each patient was, on average, eight sessions, calculated with the following formula: number of treatment sessions (i.e., 10) × number of hours per treatment session (i.e., 2) × number of therapists (i.e., 2) × number of groups (i.e., 4) divided by number of patients treated (i.e., 20). Attendance was very high across groups, with only a couple of patients missing up to two sessions, where the main reasons were being physically sick, having to attend a mandatory exam, or being on a scheduled holiday. In these instances, we tried to compensate through an individual single session, either physically or digitally. However, the occurrence of this was minimal (three single sessions).

### 2.5 Statistical procedures

Means and standard deviations from self-report measures based on completers at each assessment point are reported and constitute the foundation for calculating effect sizes using Cohen´s *d* (47) and clinical reliable change estimated according to Jacobson and Truax (48).

Statistical analyses were performed using STATA IC version 18.0. Differences in outcomes over time were assessed using planned contrasts within multilevel modeling, which is suited for non-normally distributed data and allows for the estimation of changes in repeated measures over time, despite missing data. Missing data were handled using the full information maximum likelihood estimation. All models included the fixed effects of time. In the random coefficients model for secondary outcomes, we investigated the extent to which the gains were sustained at six- and 12-month follow-ups after receiving treatment. We computed a time effect, representing the difference in change over time for the group. A linear combination of the coefficient functions was used to create model-predicted means and all planned contrasts. The model-predicted means and SE values for these variables are presented at all time points from pre-treatment to follow-up.

Means and standard deviations for self-reported perceived work ability (in the total sample) and percentage of sick leave (*n* = 14) at pre- and post-treatment are reported, and the changes were evaluated with paired-samples t-tests and described with effect sizes based on Cohen’s *d* (47). A health economic evaluation was conducted using the five dimensions of EQ-5D-5L, which constitute a health state profile and can be converted to a single utility value with three decimals. These utility values are generated from country-specific weights based on preference studies sampled from the general population (41, 49). We used a “crosswalk” technique based on the existing value-set from the UK, as suggested by the Norwegian Medicines Agency (50), as there are no established Norwegian weights. To estimate quality-adjusted life years (QALYs), utility values are multiplied by the time spent in a specific health state. These analyses were based on an intention-to-treat (ITT) sample.

## 3 Results

### 3.1 Treatment outcomes

The total diagnoses present pre- and post-treatment for the 20 patients identified by independent assessors are presented in **Table 1**. At pre-treatment, the total number of diagnoses was 45, and at post-treatment, it was 11. Only two patients still fulfilled the diagnostic criteria for a depressive episode after treatment.

**Table 1:** Frequencies of DSM-5 diagnoses at pre-treatment and post-treatment.

Diagnoses	Pre	Post
Current depressive episode	20	2
Generalized anxiety disorder	5	0
Social anxiety disorder	4	1
Unspecified eating disorder	1	0
Obsessive compulsive disorder	1	0
Attention deficit disorder	1	1
Obsessive-compulsive personality disorder	7	3
Avoidant personality disorder	5	3
Mixed personality disorder	1	1
Sum	45	11

**Table 2** reports the scores and results based on self-reports from pre- to post-treatment. Except for one patient with missing data on the SIAS and the RSE at pre-treatment and two patients missing data on the EQ-5D-5L at pre- and post-treatment, the data were complete. Based on the multilevel model analysis, all measures showed significant improvement from pre- to post-treatment. Effect sizes calculated based on completers were consistent with large effects across all measures. For the primary outcome (PHQ-9), the effect size from pre- to post-treatment, based on complete data in the total sample was 2.37.

**Table 2:** Means with standard deviations, model predicted means showing difference in change over time for the primary and secondary outcomes from pre- to post-treatment with effect sizes. .

Measure	Completers		Model predicted means ITT				*d*
	pre-treatment mean (*SD*)	Post-treatment mean (*Sd*)	Pre-treatment (se)	Post-treatment (se)	Difference in change over time Z-value (p-value)	Time effect 95% CI	
PHQ	19.45 (3.62)	7.40 (4.01)	19.71 (1.27)	5.66 (1.31)	−10.85 (0.000)	[−14.23, −9.87]	2.37
GAD	12.45 (4.61)	4.20 (2.29)	12.48 (1.26)	3.16 (1.26)	−8.38 (0.000)	[−10.18, −6.32]	1.83
BAI	17.75 (9.96)	9.25 (6.23)	16.23 (2.89)	8.23 (2.89)	−5.35 (0.000)	[−11.61, −5.39]	1.17
IIP	1.44 (0.41)	0.94 (0.39)	1.42 (0.14)	0.89 (0.14)	−5.11 (0.000)	[−.69, −.31]	1.11
SIAS	38.47 (14.13)	28.00 (14.08)	40.47 (4.87)	28.85 (4.84)	−6.25 (0.000)	[−13.85, −7.24]	1.38
MCQ	72.05 (13.23)	46.80 (8.19)	69.87 (3.73)	42.65 (3.73)	−12.11 (0.000)	[−29.34, −21.16]	2.64
POS	11.35 (4.38)	7.30 (2.00)	11.73 (1.19)	7.10 (1.19)	−5.05 (0.000)	[−5.62, −2.48]	1.10
NEG	17.10 (3.32)	9.70 (2.81)	15.56 (1.01)	8.60 (1.01)	−12.56 (0.000)	[−8.55, −6.25]	2.74
CC	14.30 (4.75)	11.10 (3.85)	14.83 (1.49)	9.90 (1.49)	−4.58 (0.000)	[−4.57, −1.83]	1.00
NC	13.90 (4.03)	7.55 (1.43)	11.94 (0.95)	6.33 (0.95)	−8.75 (0.000)	[−7.77, −4.93]	1.91
CSC	15.40 (3.68)	11.15 (2.87)	15.81 (1.15)	10.71 (1.15)	−5.51 (0.000)	[−5.76, −2.74]	1.20
RSE	8.68 (3.00)	16.16 (4.07)	8.36 (1.07)	16.98 (1.41)	8.08 (0.000)	[5.52, 9.05]	1.84
WSAS	25.55 (4.89)	11.10 (6.97)	26.81 (1.67)	9.17 (2.38)	−8.24 (0.000)	[−17.89, −11.01]	1.80
EQ	.40 (0.20)	.73 (0.13)	.31 (0.06)	.76 (0.06)	5.98 (0.000)	[.22,.44]	1.37
EQV	42.33 (17.28)	68.56 (14.44)	42.56 (5.56)	67.30 (5.56)	6.84 (0.000)	[18.71, 33.73]	1.57

SD, standard deviation; se, standard error; *d*, effect size (Cohen); PHQ, Patient Health Questionnaire; GAD, Generalized Anxiety Disorder 7; BAI, Beck Anxiety Inventory; IIP, Inventory of Interpersonal Problems; SIAS, Social Interaction Anxiety Scale; MCQ, Metacognitions Questionnaire 30; POS, positive metacognitive beliefs; NEG, negative metacognitive beliefs; CC, cognitive confidence; NC, need for control; CSC, cognitive self-consciousness; RSE, Rosenberg Self-Esteem Scale; WSAS, The Work and Social Adjustment Scale; EQ, EQ-D5-5L health related quality of life; EQV, EQ-5D-5L visual analogue scale (0–100).

Post-treatment, the Client Satisfaction Questionnaire (46) was administered to evaluate the degree of satisfaction with the intervention, and the overall level of satisfaction was very high (*M* = 27.30, *SD* = 3.63).

#### 3.1.1 Stability of treatment effects

**Figure 1:**
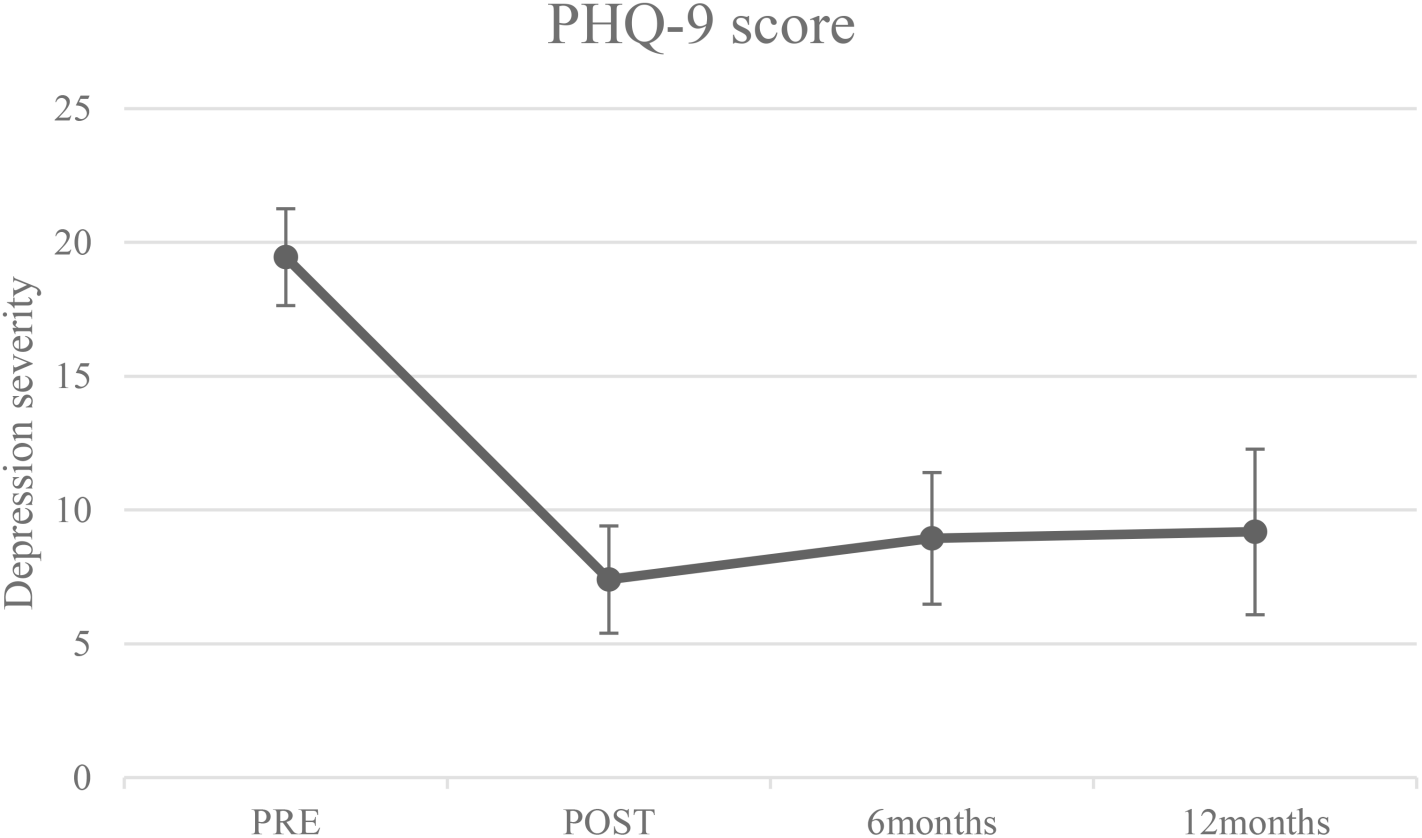
Depression severity scores (means with standard deviations) assessed with the PHQ-9 at pre-treatment, post-treatment, 6- and 12-month follow-ups for completers (n = 20 at pre- and post-treatment, and n = 17 at 6- and 12-month follow-ups).

Participants were followed up with self-report assessments at 6- and 12-month post treatments. **Table 3** reports means with standard deviations for completers, in addition to the model-predicted means and the difference in change over time based on them for the ITT sample (*N* = 20). In addition to the one patient that did not report on the SIAS and the RSE at pre-treatment, as previously mentioned, follow-up data were missing for three patients (15.0%) at the 6-month follow-up. At the 12-month follow-up, data were completely missing for three patients (15.0%), and one additional patient did not report on the BAI, IIP-32, and MCQ-30 but completed the rest of the self-report measures. Multilevel model analyses for these outcomes based on the ITT sample revealed non-significant changes over time from post-treatment through 6- and 12-month follow-ups. Except for the cognitive confidence subscale from the MCQ-30, effect sizes from pre-treatment to 6- and pre-treatment to 12-month follow-up were consistent with large effects. Regarding depression, the effect sizes from pre-treatment to the 6- and 12-month follow-ups were 1.77 and 1.75, respectively. **Figure 1** shows the mean depression severity scores with standard deviations for completers at pre-treatment, post-treatment, and 6- and 12-month follow-ups.

**Table 3:** Means with standard deviations, model predicted means showing difference in change over time for the primary and secondary outcomes from post-treatment through 6- and 12-Month follow-ups in the total ITT sample (N = 20).

Measure	Completers		Model predicted means ITT			Differences in change over time, Z-value (p-value)	Time effect 95% CI	*d* pre - 6m	*d* pre - 12m
	Mean (*SD*) 6m	Mean (*SD*) 12m	Post-treatment (se)	6m FU (se)	12m FU (se)				
PHQ	8.94 (4.92)	9.18 (6.19)	6.19 (1.37)	7.31 (1.41)	8.43 (2.01)	1.83 (0.067)	[−0.08, 2.48]	1.77	1.75
GAD	6.29 (2.91)	5.65 (3.81)	3.71 (0.83)	4.47 (0.76)	5.42 (1.17)	1.78 (0.074)	[−0.08, 1.63]	1.66	1.28
BAI	9.41 (5.81)	10.00 (6.66)	8.15 (2.05)	8.19 (1.94)	8.23 (2.11)	0.13 (0.900)	[−0.93, 1.06]	1.09	1.12
IIP	0.97 (0.39)	0.93 (0.49)	0.87 (0.12)	0.89 (0.12)	0.91 (0.18)	0.33 (0.743)	[−0.11, 0.15]	1.19	1.06
SIAS	26.18 (12.65)	25.38 (14.04)	27.76 (4.64)	26.48 (4.36)	25.21 (4.67)	−0.37 (0.711)	[−2.53, 1.73]	1.11	1.10
MCQ	50.82 (9.78)	49.06 (10.96)	43.62 (3.03)	45.06 (2.89)	46.50 (3.14)	1.74 (0.082)	[−0.16, 2.61]	1.50	1.82
POS	7.71 (2.31)	7.88 (2.22)	7.14 (0.73)	7.51 (0.69)	7.89 (0.73)	0.95 (0.340)	[−0.17, 0.50]	0.92	0.90
NEG	10.65 (2.94)	10.06 (2.54)	9.01 (0.90)	9.39 (0.75)	9.77 (0.92)	0.81 (0.419)	[−0.39, 0.95]	1.45	2.00
CC	13.41 (4.67)	11.94 (4.49)	9.98 (1.40)	10.20 (1.32)	10.43 (1.41)	1.42 (0.154)	[−0.17, 1.08]	0.38	0.63
NC	8.24 (2.11)	7.63 (1.50)	6.77 (0.50)	7.07 (0.36)	7.37 (0.54)	0.43 (0.666)	[−0.37, 0.58]	1.22	1.58
CSC	10.82 (2.70)	11.56 (3.50)	10.68 (1.03)	10.86 (0.99)	11.03 (1.04)	0.61 (0.541)	[−0.28, 0.53]	1.11	0.98
RSE	15.94 (4.22)	17.13 (5.88)	16.86 (1.40)	17.04 (1.41)	17.22 (1.80)	0.54 (0.549)	[−0.73, 1.29]	1.75	1.45
WSAS	9.82 (8.26)	10.88 (8.37)	8.34 (2.58)	8.66 (2.46)	8.97 (2.79)	0.73 (0.463)	[−0.88, 1.93]	1.49	1.31
EQ	.68 (0.21)	.71 (0.16)	0.75 (0.06)	0.73 (0.05)	0.70 (0.06)	−0.59 (0.556)	[−0.05, 0.02]	.91	1.15
EQV	67.29 (15.65)	66.47 (19.53)	67.77 (5.21)	65.08 (5.09)	62.39 (6.16)	−0.53 (0.596)	[−4.38, 2.51]	1.19	1.02

SD, standard deviation; se, standard error; FU, follow-up; PHQ, Patient Health Questionnaire; GAD, Generalized Anxiety Disorder 7; BAI, Beck Anxiety Inventory; IIP, Inventory of Interpersonal Problems; SIAS, Social Interaction Anxiety Scale; MCQ, Metacognitions Questionnaire 30; POS, positive metacognitive beliefs; NEG, negative metacognitive beliefs; CC, cognitive confidence; NC, need for control; CSC, cognitive self-consciousness; RSE, Rosenberg Self-Esteem Scale; WSAS, The Work and Social Adjustment Scale; EQ, EQ-D5-5L health related quality of life; EQV, EQ-5D-5L visual analogue scale (0–100).

#### 3.1.2 Reliable change

We evaluated the level of clinically significant change (48) using the PHQ-9 based on the completers. A reliable change was defined as a reduction of 6 points or more, with a threshold value set at 10 (e.g., 51). Individual scores at post-treatment and 6- and 12-month follow-ups were then compared to pre-treatment scores to classify patients as (1) recovered, having both a reliable improvement and a score under the cut-off; (2) improved, a clinically reliable improvement; (3) no change, indicating no clinically reliable change in any direction; and (4) worsened, indicating a clinically significant deterioration. At post-treatment, 16 of 20 patients (80%) were classified as recovered, two as improved (10%), and two as unchanged (10%). At the six-month follow up, 11 of 17 patients (65%) were classified as recovered, three as improved (18%), and three as unchanged (18%). At the 12-month follow up, 10 of 17 patients (59%) were classified as recovered, four as improved (24%), and three as unchanged (18%). None were classified as worsened at any time point.

### 3.2 Changes in self-reported work ability and status, and health-economic evaluation

At pre- and post-treatment, all participants reported their subjective work ability using the Work Ability Scale, and the 14 participants on (partial) sick leave reported their current percentage of work absence. From pre- to post-treatment, the mean subjective work ability for the total sample (*N* = 20) changed from 3.55 (*SD* = 2.35) to 6.70 (*SD* = 2.03), which indicates a strong effect (*t* = −7.018, Cohen’s *d* = 1.57). The mean sick leave percentage changed from 80.00% (*SD* = 26.89) at pre-treatment to 25.71% (*SD* = 31.31) at post-treatment (*n* = 14), indicating a strong effect in terms of return to work (RTW) (*t* = 5.529, Cohen’s *d* = 1.48). Seven of the 14 (50.0%) who were (partially) out of work pre-treatment were full-time back to work post-treatment.

Estimations of the production gain were undertaken based on the assumption of an unchanged state of self-reported sick leave for a period of 12 months in the pre-treatment state versus the equivalent assumption for the post-treatment state. The period of 12 months was selected as our follow-up data extended to 12 months, and the results indicated no change from post-treatment to 12-month follow-up. The post-treatment state was subtracted from the pre-treatment state. We used the average wage rate obtained by Statistics Norway, which was estimated to be 339 euros per day in 2016 (52, 53). The average production gain for the 20 patients over 12 months was €33,137 (95% CI [16,808–49,466]), with a total for all 20 patients of €662,743.

The incremental cost-effectiveness ratio (ICER) was calculated using a single-group pre-post design, in which the cost of the intervention was divided by the QALYs gained. The total cost of the intervention, including therapist salary and total cost of use of premises, was estimated to be €582 per participant. Health-related quality of life was measured using the EQ-5D-5L, with utility values increasing from a mean of 0.40 pre-treatment to 0.73 post-treatment. This resulted in an average gain of 0.33 QALYs. The changes in EQ-5D-5L utility values from post-treatment and over a 12-month period were not significant. The resulting incremental cost-effectiveness ratio (ICER), calculated by dividing the total costs of intervention (€582) by the gained QALY (0.33) was €1,762 per QALY gained. In Norway, there is no official fixed willingness-to-pay (WTP) threshold, but the Norwegian Directorate of Health provides guidance on the use of QALYs in economic evaluations. In the USA, the WTP threshold recommendations vary between $50,000 and $150,000 (€40,653 and 121,960) per QALY (54). In the UK, the National Institute for Health and Care Excellence has used a WTP explicit threshold between £20,000 and £30,000 (€21,932 and €32,898) per QALY gained since 2000 (55). The value from the current study is significantly lower than the commonly cited willingness-to-pay thresholds from the USA and UK per QALY gained, suggesting that the intervention may be considered cost-effective from a healthcare payer perspective.

## 4 Discussion

The effects associated with generic group MCT for individuals presenting with depression, including six- and 12-month follow-ups, were evaluated in an open trial.

The individuals treated were all diagnosed with a current moderate depressive episode but presented on average with severe depression symptoms, as indicated by their PHQ-9 scores, which averaged around 20 points at pre-treatment. A score of 16 and above is considered severe depression according to NICE (2022) (56). Moreover, comorbid disorders such as generalized anxiety disorder, social anxiety disorder, and cluster C personality disorders were frequent, and on average, each patient presented with symptoms that met the criteria for 2.25 diagnoses at pre-treatment. As a group, the patients presented with moderate anxiety, low self-esteem, and moderate interpersonal problems. General psychosocial functioning was severely affected, and 14 (70.0%) were partially or fully on sick leave due to mental illness. Eighteen (90.0%) patients had previously received other psychological treatments for depression. Hence, we argue that the sample is fairly representative of patients typically seen in specialist mental health care.

The effects associated with the treatment from pre- to post-treatment were assessed in several ways. Independent assessors evaluated the diagnostic status before and after treatment. Regarding depression, 18 of 20 patients (90.0%) no longer met the diagnostic criteria for a depressive episode post-treatment. Based on the PHQ-9, the mean level of improvement in depression symptoms was consistent with a large effect size (*d =* 2.37), and in evaluating clinically significant change, 16 (80.0%) were classified as recovered, two (10.0%) as improved, and two (10.0%) as unchanged. Analysis of change over time using an ITT sample indicated stability in terms of depression scores from post-treatment through six- and 12-month follow-ups (indicated by a non-significant time effect). However, the effect sizes from pre-treatment to six- and 12-month follow-ups (based on completers) were reduced, but still consistent with large effects, and clinically significant change classifications indicated that recovery rates sank from 80% to 65% and 59% (again, based on completers). However, relative to post-treatment, only one more patient (from two to three) was classified as unchanged at follow-up points, indicating stability in reliable improvement from post-treatment to 12 months after treatment.

Regarding secondary outcomes, the independent assessors identified 25 comorbid disorders at pre-treatment for the sample, which was reduced to nine at post-treatment. Furthermore, the treatment was associated with substantial improvements and large effects on various anxiety symptoms (including generalized and social interaction anxiety), interpersonal problems, self-esteem, general functioning, and quality of life. Dysfunctional metacognitive beliefs substantially improved, as indicated by large effect sizes, with negative metacognitive beliefs changing the most. As with depression symptoms, changes over time for secondary outcomes from post-treatment through six- and 12-month follow-ups were non-significant, indicating stability at the group level. However, the effect sizes from pre-treatment to follow-up points based on completers were somewhat lower than the effect sizes from pre- to post-treatment, which is likely linked to more individual variability in the scores at these time points.

These results are in line with previous studies on MCT for depression, reporting substantial recovery rates and strong effects (see 10 for a review), as well as support for MCT’s transdiagnostic and trans-symptomatic effect (17, 18). Interestingly, treatment was associated with the loss of several comorbid diagnoses, including personality disorders. This observation is consistent with a previous study on individual MCT for depression (21). Reducing the CAS and its underlying biases in metacognitive beliefs might not only impact comorbid emotional disorders but also improve personality problems. We note that both self-esteem and interpersonal distress significantly improved, showing large effects, which supports this notion and is consistent with observational longitudinal studies indicating a role for dysfunctional metacognitive beliefs in personality functioning (16). Furthermore, the strong and broad effects are consistent with Wells’ (9) idea that metacognitive change is a universal mechanism of change, in which modifying negative metacognitive beliefs is seen as particularly important (see 57 for a review), as it will reduce the CAS and thus enable reflexive and adaptive self-regulation.

Among those on (partial) sick leave at pre-treatment (*n* = 14), half had fully returned to work at post-treatment, and the total sick leave percentage was reduced from approximately 80% to 26%, consistent with a large effect size of 1.48. In addition, perceived work ability substantially improved in the total sample, marked by an average of a three-point shift on the work ability scale and an effect size of 1.57. This is a promising shift, as the single-item WAS has predictive validity for long-term sick leave (58, 59). This result is also consistent with a recent study on the attention training technique (an MCT technique; 9) delivered in a group format, showing positive effects on WAS (60). Interestingly, MCT is associated with improved work ability and status, as the treatment has no explicit focus on work-related problems. A likely explanation is that dysfunctional metacognitive beliefs play a role in poor work ability (11) and work absence (61, 62) implying that modifying these metacognitive beliefs can improve work-related psychological dysfunction and contribute to returning to work. Consistent with our findings, others have also reported that individual MCT was associated with positive effects on work status and usage of disability pensions in patients treated for major depressive disorder (63) and that MCT was more effective than wait-list control for RTW in patients with common mental disorders (64).

Meta-analytic evidence suggests that high relapse rates are the norm rather than the exception for patients receiving standard care (6). The metacognitive model suggests that stability and relapse following treatment are related to remaining or re-emerging biases in metacognition (8). The effects associated with the treatment in this trial were substantial from pre- to post-treatment and corresponded to large improvements in dysfunctional metacognitive beliefs, with the strongest observed effect in negative metacognitive beliefs, which is highlighted as the most important domain in metacognitive theory (9). While changes over time from post-treatment through six- and 12-month follow-ups at the group level indicated stable effects, there were also indicators of re-emerging symptoms at these time points, e.g., based on the number of patients classified as recovered, which suggests individual variation. Similar observations are valid for metacognitive belief domains, where the effect sizes were lower at follow-up points than post-treatment. Hence, the proposed mechanisms and clinical outcomes appear to co-vary; however, we cannot use the current data to understand these relationships in more detail. While recovery rates of 60% at the 12-month follow-up are very good, they are slightly lower than that reported from trials on individual MCT for depression (e.g., 73% of completers; 65). There can be several explanations for this difference, including the treatment format (i.e., individual versus group), setting (i.e., university outpatient clinic versus specialist mental health care setting), use of a generic rather than a disorder-specific metacognitive approach (compromising specificity), or sample characteristics.

With limited health resources and increasing demands for psychological treatments, the question of intervention cost-effectiveness is more relevant than ever. Therefore, we conducted a health economic assessment, which not only yielded clinically meaningful improvements in health-related quality of life but also demonstrated strong economic value from a healthcare payer perspective. The estimated production gains for the 20 patients were €662,743 over 12 months. Importantly, the calculated incremental cost-effectiveness ratio (ICER) of €1,762 per QALY gained is substantially below the commonly accepted willingness-to-pay (WTP) thresholds in countries such as the UK (£20,000–£30,000) and the USA ($50,000–$150,000). These results indicate that the intervention is highly cost-effective, particularly given the modest per-participant cost of €582 and sustained improvement in EQ-5D-5L utility scores over the 12-month follow-up. Although no separate WTP threshold exists specifically for mental health disorders in Norway, the severity and chronicity of such conditions may justify higher thresholds, further strengthening the case for investment in generic group MCT in this setting. We note that the treatment was delivered by highly trained therapists, which is crucial when evaluating a treatment’s potential. Simultaneously, the scalability of the intervention will depend on substantial investments in therapist training and supervision, which should be considered as part of a larger cost-effectiveness evaluation.

Our study has several strengths. The treatment was conducted in a naturalistic setting and was delivered by trained MCT therapists. Participants underwent an assessment of clinical diagnoses at pre-treatment by independent assessors who were clinical psychologists, and the patients’ diagnoses were reassessed at post-treatment. A range of symptom domains and psychological difficulty areas were assessed, which speaks to the transdiagnostic nature of MCT. However, several important limitations must be considered. The uncontrolled design means that we cannot partially out the effects of treatment from other influences on symptoms (e.g., therapist effects). The authors of this manuscript also conducted the treatment, so there are dual roles. Participants were followed up at 6 and 12 months with self-report measures, but no clinical assessment or other contact was made as the patients were discharged from the service. Reliability of the clinical diagnostic assessments was not formally conducted, and assessment of adherence and competence was not conducted. The health economic evaluation was based on a small sample size and self-report rather than, for example, registry data. Future research should include patients’ use of additional healthcare services to fully capture the value of mental health interventions.

In conclusion, generic group MCT delivered in a specialist mental health care setting for primary depression was resource-efficient, associated with an 80% recovery rate from depression, and had a strong and broad effect across secondary outcomes at post-treatment. Treatment gains were largely maintained through the six- and 12-month follow-ups. These results warrant a larger randomized controlled trial of generic group MCT, including a control group and ideally longer follow-up that is not restricted to self-report. However, the findings suggest that generic group MCT is promising even in naturalistic settings at the highest level of specialist mental health services and that it is likely a cost-effective intervention.

## Data Availability

The datasets presented in this article are not readily available because In line with the ethical approval, the data is not available in order to protect the privacy of the participants. Requests to access the datasets should be directed to HN, henrik.nordahl@ntnu.no.

## References

[1] Norwegian Institute of Public Health . Psykiske lidelser hos voksne. (2021). Available online at: https://www.fhi.no/nettpub/hin/psykisk-helse/psykiske-lidelser-voksne/ (Accessed January 5, 2025).

[2] Herrman H Patel V Kieling C Berk M Buchweitz C Cuijpers P . Time for united action on depression: a Lancet–World Psychiatric Association Commission. Lancet. (2022) 399:957–1022. doi: 10.1016/S0140-6736(21)02141-3, PMID: 35180424

[3] Saha S Lim CC Cannon DL Burton L Bremner M Cosgrove P . Co-morbidity between mood and anxiety disorders: A systematic review and meta-analysis. Depression Anxiety. (2021) 38:286–306. doi: 10.1002/da.23113, PMID: 33225514 PMC7984258

[4] Friborg O Martinsen EW Martinussen M Kaiser S Øvergård KT Rosenvinge JH . Comorbidity of personality disorders in mood disorders: a meta-analytic review of 122 studies from 1988 to 2010. J Affect Disord. (2014) 152:1–11. doi: 10.1016/j.jad.2013.08.023, PMID: 24120406

[5] Amiri S Behnezhad S . Depression symptoms and risk of sick leave: a systematic review and meta-analysis. Int Arch Occup Environ Health. (2021) 94:1495–512. doi: 10.1007/s00420-021-01703-0, PMID: 33928429

[6] Cuijpers P Miguel C Harrer M Ciharova M Karyotaki E . The outcomes of mental health care for depression over time: a meta-regression analysis of response rates in usual care. J Affect Disord. (2024) 358:89–96. doi: 10.1016/j.jad.2024.05.019, PMID: 38710332

[7] Wells A Matthews G . Attention and Emotion: A clinical perspective. Hove UK: Erlbaum (1994).

[8] Wells A . Breaking the cybernetic code: Understanding and treating the human metacognitive control system to enhance mental health. Front Psychol. (2019) 10:2621. doi: 10.3389/fpsyg.2019.02621, PMID: 31920769 PMC6920120

[9] Wells A . Metacognitive therapy for anxiety and depression. New York: Guilford press (2009).

[10] Wells A Nordahl H . Metacognition and mental regulation. In: Dozois DJA Dobson KS , editors. Treatment of psychosocial risk factors in depression. Washington, DC: American Psychological Association (2023). p. 383–406.

[11] Anyan F Hjemdal O Nordahl H . Testing the longitudinal effect of metacognitive beliefs on the trajectory of work ability. Curr Psychol. (2023) 42:28086–94. doi: 10.1007/s12144-022-03912-3

[12] Cano-López JB Anyan F García-Sancho E Nordahl H Salguero JM . A within-person test of the metacognitive model: Daily dynamics between metacognitive beliefs, metacognitive strategies, and negative affect. J Anxiety Disord. (2024) 107:102930. doi: 10.1016/j.janxdis.2024.102930, PMID: 39305537

[13] Hoffart A Skjerdingstad N Freichel R Johnson SU Epskamp S Ebrahimi OV . Mapping the dynamics of generalized anxiety symptoms and actionable transdiagnostic mechanisms: A panel study. Depression Anxiety. (2025) 2025:1885004. doi: 10.1155/da/1885004, PMID: 40395980 PMC12092150

[14] Nordahl H Anyan F Hjemdal O . Prospective relations between dysfunctional metacognitive beliefs, metacognitive strategies, and anxiety: Results from a four-wave longitudinal mediation model. Behav Ther. (2023) 54:765–76. doi: 10.1016/j.beth.2023.02.003, PMID: 37597956

[15] Solheim M Pukstad E Anyan F Strand ER Nordahl H . Is mental regulation related to self-esteem? Testing a basic metacognitive model. Curr Psychol. (2024) 43:21208–17. doi: 10.1007/s12144-024-05892-y

[16] Strand ER Anyan F Hjemdal O Nordahl HM Nordahl H . Metacognitive beliefs prospectively predict level of personality functioning beyond maladaptive personality traits within-individuals: Results from a four-wave longitudinal study. Pers Individ Dif. (2024) 230:112812. doi: 10.1016/j.paid.2024.112812

[17] Andersson E Aspvall K Schettini G Kraepelien M Särnholm J Wergeland GJ . Efficacy of metacognitive interventions for psychiatric disorders: a systematic review and meta-analysis. Cogn Behav Ther. (2025) 54:276–302. doi: 10.1080/16506073.2024.2434920, PMID: 39692039

[18] Normann N Morina N . The efficacy of metacognitive therapy: A systematic review and meta-analysis. Front Psychol. (2018) 9:2211. doi: 10.3389/fpsyg.2018.02211, PMID: 30487770 PMC6246690

[19] Hagen R Hjemdal O Solem S Kennair LEO Nordahl HM Fisher P . Metacognitive therapy for depression in adults: a waiting list randomized controlled trial with six months follow-up. Front Psychol. (2017) 8:31. doi: 10.3389/fpsyg.2017.00031, PMID: 28174547 PMC5258745

[20] Callesen P Reeves D Heal C Wells A . Metacognitive therapy versus cognitive behaviour therapy in adults with major depression: a parallel single-blind randomised trial. Sci Rep. (2020) 10:7878. doi: 10.1038/s41598-020-64577-1, PMID: 32398710 PMC7217821

[21] Hjemdal O Hagen R Solem S Nordahl H Kennair LEO Ryum T . Metacognitive therapy in major depression: an open trial of comorbid cases. Cogn Behav Pract. (2017) 24:312–8. doi: 10.1016/j.cbpra.2016.06.006

[22] Winter L Gottschalk J Nielsen J Wells A Schweiger U Kahl KG . A comparison of metacognitive therapy in current versus persistent depressive disorder – a pilot outpatient study. Front Psychol. (2019) 10:1714. doi: 10.3389/fpsyg.2019.01714, PMID: 31447722 PMC6691034

[23] Groves SJ Porter RJ Jordan J Knight R Carter JD McIntosh VV . Changes in neuropsychological function after treatment with metacognitive therapy or cognitive behavior therapy for depression. Depression Anxiety. (2015) 32:437–44. doi: 10.1002/da.22341, PMID: 25677736

[24] Dammen T Papageorgiou C Wells A . An open trial of group metacognitive therapy for depression in Norway. Nordic J Psychiatry. (2015) 69:126–31. doi: 10.3109/08039488.2014.936502, PMID: 25124119

[25] Papageorgiou C Wells A . Group metacognitive therapy for severe antidepressant and CBT resistant depression: A baseline-controlled trial. Cogn Ther Research 39. (2015) 39:14–22. doi: 10.1007/s10608-014-9632-x

[26] Maj M Stein DJ Parker G Zimmerman M Fava GA De Hert M . The clinical characterization of the adult patient with depression aimed at personalization of management. World Psychiatry. (2020) 19:269–93. doi: 10.1002/wps.20771, PMID: 32931110 PMC7491646

[27] Callesen P Capobianco L Heal C Juul C Find Nielsen S Wells A . A preliminary evaluation of transdiagnostic group metacognitive therapy in a mixed psychological disorder sample. Front Psychol. (2019) 10:1341. doi: 10.3389/fpsyg.2019.01341, PMID: 31281277 PMC6595247

[28] Capobianco L Reeves D Morrison AP Wells A . Group metacognitive therapy vs. mindfulness meditation therapy in a transdiagnostic patient sample: a randomised feasibility trial. *Psychiatry Research*. (2018) 259:554–61. doi: 10.1016/j.psychres.2017.11.045, PMID: 29179137

[29] Strand ER Veium LT Engvik LS Nordahl H . Generic group metacognitive therapy for patients with major depressive disorder and related problems: A preliminary evaluation in specialized mental health care. Int J Cogn Ther. (2023) 16:497–509. doi: 10.1007/s41811-023-00175-z

[30] First MB Williams JB Benjamin LS Spitzer RL . User's guide for the structured clinical interview for DSM-5® personality disorders (SCID-5-PD). Arlington, VA: American Psychiatric Association Publishing (2016).

[31] First MB Williams JB Karg RS Spitzer RL . User's guide for the SCID-5-CV Structured Clinical Interview for DSM-5® disorders: Clinical version. Washington, DC: American Psychiatric Publishing, Inc (2016).

[32] American Psychiatric Association . Diagnostic and statistical manual of mental disorders, 5th ed. Washington DC (2013). doi: 10.1176/appi.books.9780890425596.

[33] Kroenke K Spitzer RL Williams JB . The PHQ-9: validity of a brief depression severity measure. J Gen Internal Med. (2001) 16:606–13. doi: 10.1046/j.1525-1497.2001.016009606.x, PMID: 11556941 PMC1495268

[34] Spitzer RL Kroenke K Williams JB Löwe B . A brief measure for assessing generalized anxiety disorder: the GAD-7. Arch Internal Med. (2006) 166:1092–7. doi: 10.1001/archinte.166.10.1092, PMID: 16717171

[35] Beck AT Epstein N Brown G Steer RA . An inventory for measuring clinical anxiety: psychometric properties. J Consulting Clin Psychol. (1988) 56:893. doi: 10.1037/0022-006X.56.6.893, PMID: 3204199

[36] Mattick RP Clarke JC . Development and validation of measures of social phobia scrutiny fear and social interaction anxiety. Behav Res Ther. (1998) 36:455–70. doi: 10.1016/S0005-7967(97)10031-6, PMID: 9670605

[37] Barkham M Hardy GE Startup M . The IIP-32: A short version of the Inventory of Interpersonal Problems. Br J Clin Psychol. (1996) 35:21–35. doi: 10.1111/j.2044-8260.1996.tb01159.x, PMID: 8673033

[38] Mundt JC Marks IM Shear MK Greist JM . The Work and Social Adjustment Scale: a simple measure of impairment in functioning. Br J Psychiatry. (2002) 180:461–4. doi: 10.1192/bjp.180.5.461, PMID: 11983645

[39] Rosenberg M . Society and the adolescent self-image. Princeton: Princeton University Press (1965). doi: 10.1515/9781400876136

[40] Wells A Cartwright-Hatton S . A short form of the metacognitions questionnaire: properties of the MCQ-30. Behav Res Ther. (2004) 42:385–96. doi: 10.1016/s0005-7967(03)00147-5, PMID: 14998733

[41] Herdman M Gudex C Lloyd A Janssen MF Kind P Parkin D . Development and preliminary testing of the new five-level version of EQ-5D (EQ-5D-5L). Qual Life Res. (2011) 20:1727–36. doi: 10.1007/s11136-011-9903-x, PMID: 21479777 PMC3220807

[42] Feng Y-S Kohlmann T Janssen MF Buchholz I . Psychometric properties of the EQ-5D-5L: a systematic review of the literature. Qual Life Res. (2021) 30:647–73. doi: 10.1007/s11136-020-02688-y, PMID: 33284428 PMC7952346

[43] Garratt AM Hansen TM Augestad LA Rand K Stavem K . Norwegian population norms for the EQ-5D-5L: results from a general population survey. Qual Life Res. (2022) 31:517–26. doi: 10.1007/s11136-021-02938-7, PMID: 34272631 PMC8284681

[44] Ilmarinen J Tuomi K Eskelinen L Nygård CH Huuhtanen P Klockars M . Summary and recommendations of a project involving cross-sectional and follow-up studies on the aging worker in Finnish municipal occupations, (1981—1985). Scandinavian J Work Environ Health. (1991) 17:135–41. Available online at: https://www.jstor.org/stable/40965955 (Accessed March 24, 2025)., PMID: 1792527

[45] Tuomi K Ilmarinen J Eskelinen L Järvinen E Toikkanen J Klockars M . Prevalence and incidence rates of diseases and work ability in different work categories of municipal occupations. Scandinavian J Work Environ Health. (1991) 17:67–74. Available online at: https://www.jstor.org/stable/40965945 (Accessed March 24, 2025)., PMID: 1792531

[46] Nguyen TD Attkisson CC Stegner BL . Assessment of patient satisfaction: development and refinement of a service evaluation questionnaire. Eval Program Plann. (1983) 6:299–313. doi: 10.1016/0149-7189(83)90010-1, PMID: 10267258

[47] Cohen J . Statistical power analysis for the behavioral sciences. New York, NY: Routledge Academic (1988).

[48] Jacobson NS Truax P . Clinical significance: a statistical approach to defining meaningful change in psychotherapy research. J Consulting Clin Psychol. (1991) 59:12–9. doi: 10.1037/0022-006X.59.1.12 2002127

[49] EuroQol Research Foundation . EQ-5D-5L User Guide Version 3.0: Basic information on how to use the EQ-5D-5L instrument. (2019). Available online at: https://euroqol.org/publications/user-guides (Accessed September 6, 2025).

[50] Statens legemiddelverk . Guidelines for the submission of documentation for single technology assessment (STA) of pharmaceuticals - Legemiddelverket. Statens legemiddelverk (2021). Available online at: https://legemiddelverket.no/english/public-funding-and-pricing/documentation-for-sta/guidelines-for-the-submission-of-documentation-for-single-technology-assessment-sta-of-pharmaceuticals (Accessed March 03, 2025).

[51] Gyani A Shafran R Layard R Clark DM . Enhancing recovery rates: lessons from year one of IAPT. Behav Res Ther. (2013) 51:597–606. doi: 10.1016/j.brat.2013.06.004, PMID: 23872702 PMC3776229

[52] Aasdahl L Fimland MS Bjørnelv GMW Gismervik SØ. Johnson R Vasseljen O . Economic evaluation of impatient multimodal occupational rehabilitation vs. outpatient acceptance and commitment therapy for sick-listed workers with musculoskeletan- or common mental disorders. J Occup Rehabil. (2022) 33:463–72. doi: 10.1007/s10926-022-10085-0, PMID: 36949254 PMC10495483

[53] Statistics Norway . Wages – Occupational monthly salary, by sector, sex and working hours 2015–2021. Available online at: https://www.ssb.no/statbank/table/11418 (Accessed September 6, 2025).

[54] Neumann PJ Kim DD . Cost-effectiveness thresholds used by study authors 1990-2021. JAMA. (2023) 329:1312–4. doi: 10.1001/jama.2023.1792, PMID: 37071104 PMC10114019

[55] Bertram MY Lauer JA De Joncheere K Edejer T Hutubessy R Kieny MP . Cost–effectiveness thresholds: pros and cons. Bull World Health Organ. (2016) 94:925–30. doi: 10.2471/BLT.15.164418, PMID: 27994285 PMC5153921

[56] National Institute for Health and Care Excellence (NICE) . Depression in adults: treatment and management. (2022). Available online at: https://www.nice.org.uk/guidance/ng222 (Accessed January 5, 2025). 35977056

[57] Pukstad E Halvorsen JØ. Jensen MR Solhaug S Nordahl H . Do metacognitive beliefs satisfy criteria as mechanisms of change in treatment? A systematic review and evidence synthesis. Clin Psychol Rev. (2025) 122:102654. doi: 10.1016/j.cpr.2025.102654, PMID: 41056592

[58] Kinnunen U Nätti J . Work ability score and future work ability as predictors of register-based disability pension and long-term sickness absence: a three-year follow-up study. Scandinavian J Public Health. (2018) 46:321–30. doi: 10.1177/1403494817745190, PMID: 29212430

[59] Lundin A Leijon OLA Vaez M Hallgren M Torgén M . Predictive validity of the Work Ability Index and its individual items in the general population. Scandinavian J Public Health. (2017) 45:350–6. doi: 10.1177/1403494817702759, PMID: 28385066

[60] Snuggerud TR Nordahl H Vrabel K Hoffart A Johnson SU . The feasibility and preliminary outcomes of a two-week attention training technique intervention for young adults with mixed anxiety disorders. Cogn Behav Ther. (2025), 1–19. doi: 10.1080/16506073.2025.2509168, PMID: 40424353

[61] Nordahl H Wells A . In or out of work: A preliminary investigation of mental health, trait anxiety and metacognitive beliefs as predictors of work status. Clin Psychol. (2019) 23:79–84. doi: 10.1111/cp.12153

[62] Nordahl H Wells A . Social anxiety and work status: the role of negative metacognitive beliefs, symptom severity and cognitive-behavioural factors. J Ment Health. (2020) 29:665–9. doi: 10.1080/09638237.2017.1340622, PMID: 28648099

[63] Solem S Kennair LEO Hagen R Havnen A Nordahl HM Wells A . Metacognitive therapy for depression: A 3-year follow-up study assessing recovery, relapse, work force participation, and quality of life. Front Psychol. (2019) 10:2908. doi: 10.3389/fpsyg.2019.02908, PMID: 31920902 PMC6936246

[64] Gjengedal RG Hannisdal M Osnes K Reme SE Wells A Blonk R . Metacognitive therapy and work-focus for patients with depression, anxiety or comorbid depression and anxiety on sick leave: a single-centre, open-label randomised controlled trial. eClinicalMedicine. (2025) 89:103613. doi: 10.1016/j.eclinm.2025.103613, PMID: 41357335 PMC12675033

[65] Hjemdal O Solem S Hagen R Kennair LEO Nordahl HM Wells A . A randomized controlled trial of metacognitive therapy for depression: Analysis of 1-year follow-up. *Frontiers in Psychology*. 10. (2019) 1842. doi: 10.3389/fpsyg.2019.01842, PMID: 31440193 PMC6694776

